# Construction and application of service quality evaluation system in the preclinical research on cardiovascular implant devices

**DOI:** 10.1186/s12911-019-0773-4

**Published:** 2019-02-28

**Authors:** Yongchun Cui, Fuliang Luo, Boqing Yang, Bin Li, Qi Zhang, Gopika Das, Guangxin Yue, Jiajie Li, Yue Tang, Xin Wang

**Affiliations:** 1 Animal Experimental Center, Fuwai Hospital, Chinese Academy of Medical Sciences and Peking Union Medical College, State Key Laboratory of Cardiovascular Disease & Center for cardiovascular experimental study and evaluation, National Center for Cardiovascular Diseases, Beijing Key Laboratory of Pre-clinical Research and Evaluation for Cardiovascular Implant Materials, Beijing, 100037 China; 2 Department of Biology, Boston University, Boston, MA 02215 USA; 3 School of Agricultural Economics and Rural Development, Renmin University of China, Beijing, 100872 China

**Keywords:** Fuzzy analytical hierarchy process, Cardiovascular implant devices, Preclinical research and development service, Quality evaluation system

## Abstract

**Background:**

Services for the preclinical development and evaluation of cardiovascular implant devices (CVIDs) is a new industry. However, there is still no indicator system for quality evaluation. Our aim is to construct a service for quality evaluation system for the preclinical research and development of CVIDs based on Fuzzy Analytical Hierarchy Process (FAHP).

**Methods:**

First, we reviewed the related literature to identify and select possible factors. Second, we developed an analytic hierarchy process framework. Third, we developed a questionnaire based on pairwise comparisons and invited 10 experienced specialists to rate these factors. We then used FAHP to compute the weights of these factors and prioritize them. Finally, to demonstrate the effectiveness of the proposed indicator system, a case study was performed as a practical example.

**Results:**

Four main indicators (professionalism, functionality, stability and security) and 15 subindicators were selected to form the service evaluation system based on literature review and expert’s proposals. According to the weight calculation data, the order of primary indicators by importance, is professionalism (0.6457), security (0.1193), functionality (0.0958) and stability (0.0596) in sequence. Top five secondary indices are personnel’s technical ability, facility and equipment attractiveness, data auditability, confidentiality capability and professional service procedures. In the case study, FW’s final actual effectiveness value was 0.9076, which is the same as the actual situation.

**Conclusion:**

The indicator system established in this study is comprehensive, reasonable, reliable and with strong practicality. It is worth popularizing and applying. The implementation of this evaluation system can provide measurable evidence for service demander and a way to improve service quality for suppliers.

**Electronic supplementary material:**

The online version of this article (10.1186/s12911-019-0773-4) contains supplementary material, which is available to authorized users.

## Background

Cardiovascular disease is the leading cause of death and disability worldwide [1–4]. Implantation of high-quality medical devices is one of the most commonly used means for the treatment of cardiovascular diseases. With the increasing morbidity and mortality of cardiovascular disease, there is an increasing market demand for high-quality cardiovascular implant devices (CVIDs), such as coronary artery stent, heart valves and artificial assistant equipment [5–8].

CVIDs belong to high-risk class III medical devices, which are characterized by security primacy, technical complexity, professional operation, long development line and high cost. Therefore, the need for a new service industry for the preclinical development and evaluation of implant devices arises at this historic moment. However, there is still no service quality evaluation system, which might be the first step to the smooth, efficient running and standardization of this new industry [9].

According to previous studies, Analytic Hierarchy Process is a useful method for analysis of factors influencing service [10], and medical decision support [11–14]. The fuzzy theory has also been recommended for its ability to prevent expert judgment from being influenced by extreme values, to combine the participants’ opinions more reasonably, as well as both objectively and accurately to prioritize the relevant indicators and to calculate their weight values under a hierarchy model [15–18]. This suggests to us that the Fuzzy Analytic Hierarchy Process (FAHP) has the advantages of both AHP and fuzzy theory, and it maybe an effective method for establishing a service quality evaluation system for the preclinical development of CVIDs [19–21].

In the present study, we will focus on establishing a service quality evaluation system for the preclinical development of cardiovascular implant devices, based on FAHP. This would contribute to strengthening quality control and regulating the behavior in preclinical research and development of medical device, so as to guarantee that the public can safely and effectively use them [22, 23].

## Methods

### Arrangement of a decision-making group (DMG)

First, a DMG was organized to outline a structure for the process indicators according to the previously reported inclusive criteria [24, 25] as follows:

1. Having an academic degree (PhD, MSc) in one of the mentioned majors: clinical, management.
2. Working as faculty in a hospital or Research Institute, associated with cardiovascular implant devices.
3. Having empirical studies in subjects relevant to cardiovascular device development and application.
4. willing to answer the expert consultation form

### Establishment of the evaluation framework

Then, we comprehensively reviewed literature [26–31] and collected the comments of the DMG. Indicators that could most effectively reflect the service quality in the preclinical development of cardiovascular implant devices were selected for constructing the two- index-hierarchy indicator system. The primary dimensions included professionalism, security, functionality and stability. The secondary dimensions included personnel’s technical ability, hardware attractiveness, professional service procedures, permission suitability, confidentiality capability of information and resources, etc.

### Calculation of the weights and ranks of the indicators

#### Construct pair-wise comparison (PWC) matrix

With regard to the service validity evaluation framework, the DMG were required to perform pair-wise comparisons between the first and second levels of indicators, on the basis of their knowledge. To do this, they compared the importance of each indicator of both the first and second levels, with the adjacent indicators of its own level. Then, a matrix^−^*X* was created according to the pair-wise comparisons [32–34].

1 $$ {\tilde{x}}_{ij}=\left[\begin{array}{cccc}{\tilde{x}}_{11}& {\tilde{x}}_{12}& \cdots & {\tilde{x}}_{1n}\\ {}{\tilde{x}}_{21}& {\tilde{x}}_{22}& \cdots & {\tilde{x}}_{2n}\\ {}\vdots & \vdots & \ddots & \vdots \\ {}{\tilde{x}}_{n1}& {\tilde{x}}_{n2}& \cdots & {\tilde{x}}_{nn}\end{array}\right] $$

#### Comparison matrix consistency check

After the comparison, matrices were established, and the consistency checks of the matrices was performed by computing the consistency ratio (CR):

2 $$ \mathrm{CR}=\frac{\mathrm{CI}}{\mathrm{RI}};\mathrm{CI}=\left({\uplambda}_{max}-n\right)/\left(n-1\right) $$

Where: *λmax* is the largest Eigen value of the comparison matrix. “*CI*” indicates the consistency index,“*RI*” denotes the random index, and “*n*” is the number of criteria that would be judged against (i.e., matrix size).

#### Determination of the indicators’ weights

Linguistic variables are used in the questionnaire to convert the measured qualitative factors to fuzzy numbers (see Additional file 1). The linguistic variables chosen are commonly used variables - equally important (EI), weakly more important (WMI), strongly more important (SMI), very strongly more important (VSMI), and absolutely more important (AMI). To score the importance of indicators influencing the service quality for preclinical research on cardiovascular implant devices, 1/9–9 scaling method was used as the scoring principle which showed the relative importance of the former indicator (A) compared with the latter indicator (B). All of the primary indicators and the secondary indicators were paired and compared respectively. The data were shown in Additional file 2.

The pair-wise comparison matrix between criteria is then formed based on the fuzzy numbers to evaluate the weights using the FAHP method [35–39]. Based on the previously constructed pair-wise comparison matrix^−^*xij*, the weights determined are as follows [24]:

3 $$ {\alpha}_j{\left[\underset{j=1}{\overset{n}{\Pi}}{l}_{ij}\right]}^{1/n};{\beta}_j={\left[\underset{j=1}{\overset{n}{\Pi}}{m}_{ij}\right]}^{1/n};{\gamma}_j{\left[\underset{j=1}{\overset{n}{\Pi}}{n}_{ij}\right]}^{1/n};{\delta}_j={\left[\underset{j=1}{\overset{n}{\Pi}}{s}_{ij}\right]}^{1/n} $$

and

4 $$ \alpha =\underset{j=1}{\overset{n}{\Sigma}}{\alpha}_j;\beta =\underset{j=1}{\overset{n}{\Sigma}}{\beta}_j;\gamma =\underset{j=1}{\overset{n}{\Sigma}}{\gamma}_j;\delta =\underset{j=1}{\overset{n}{\Sigma}}{\delta}_j $$

We then prioritized the extracted indicators of service quality in a hierarchy model identified by the FAHP approach [4] (see Additional file 3).

## Results

### Demographic characteristics of experts

A total of 10 experts with a senior professional title in the internal (*n* = 5) and surgery (n = 5) departments of national center for cardiovascular diseases, were invited to participate in this study. Half male and half female, most belonged to the 30–50 age group. Six expert’s work experience was 5–10 years (60%), 3 expert’s work experience was 10–20 years (30%) and 1 expert’s work was more than 20 years (10%). Information describing the DMs is presented in Table 1.

**Table 1:** Descriptive demographic characteristics of specialists

Item		Number of responders	Percentage (%)
Gender	Male	5	50
	Female	5	50
Age	30–40	4	40
	40–50	4	40
	More than 50	2	20
Work experience	5–10	6	60
	10–20	3	30
	20–30	1	10
Professional title	Professor	8	80
	Associate professor	2	20

### Extracting the affecting dimensions of service quality

After a comprehensive review of the literature [17, 19, 21] and consideration of the DMG’s opinions, we selected indicators that can be applied to effectively assess the service quality in preclinical development of cardiovascular implant devices. Thus a two-level evaluation system, including primary and secondary dimensions, was established.

**Fig. 1:**
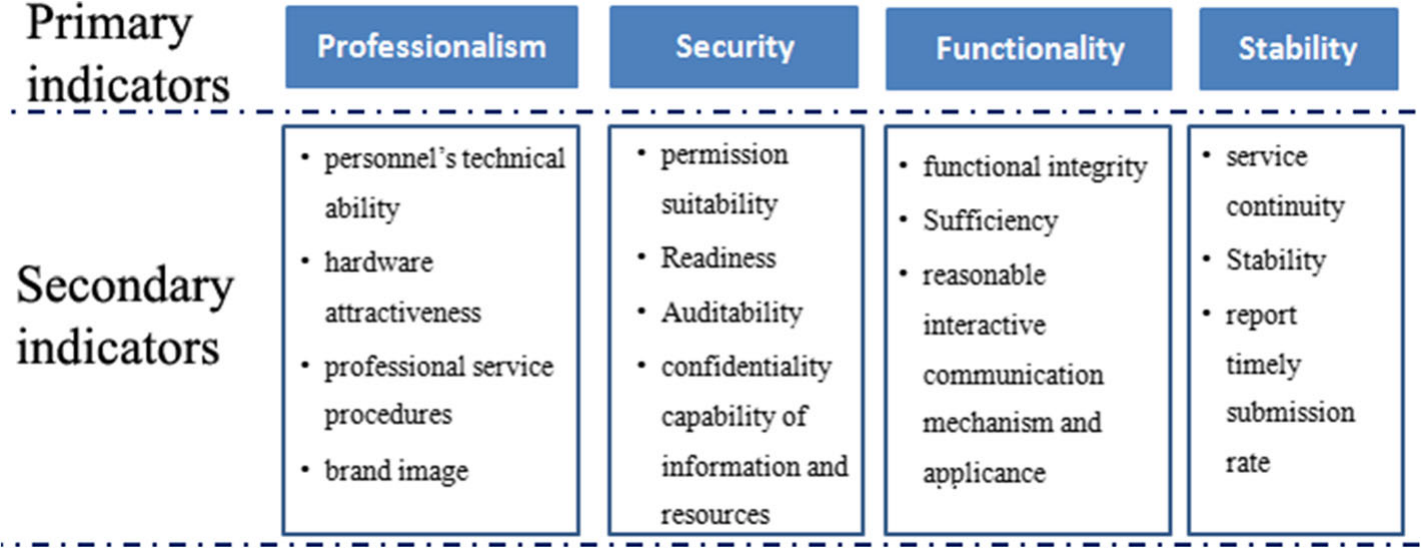
The two-level evaluation system. Based on literature review and DMG’s opinions, the two-level evaluation system was established. The first level indicator includes 4 items, and the second level indicator includes 15 items. DMG means decision-making group

As shown in Table 2 and Fig. 1, the primary dimensions include professionalism, security, functionality and stability. With respect to the DMG expertise, we further developed some secondary indicators for each primary dimension. “Professionalism” indicators include personnel’s technical ability, hardware attractiveness, professional service procedures and brand image. Security includes the permission suitability, readiness, auditability and confidentiality capability of information and resources. Functionality includes functional integrity, sufficiency, reasonable interactive communication mechanism and applicance. Stability includes service continuity, stability, and report timely submission rate.

**Table 2:** Hierarchical structure of indicators and sub-indicators

Primary indicator	Sub- indicator	Definition
Professionalism B1	Brand image C11	1. Qualified or not 2. The level of approved qualifications 3. The ranking among government, industry, manufacturers and users and the market share
	Personnel’s technical ability C12	1. The ratio of the number of qualified technical personnel and the total number of technical personnel 2. The ratio of technical personnel with bachelor degree or above and the total number of technical personnel.
	Facility and hardware attractiveness C13	1. Use equipments or not 2. Use equipments in part or full course of project service. And the matched-degree between the tool and theproject task.
	Professional service procedures C14	1. Whether is a documented or automated service process established and how is it implemented 2. Whether achieved ISO/IEC 20000 certification and how is it implemented
FunctionalityB2	Functional integrity C21	Ratio of the actual number of functions implemented to the number of functions agreed in the service contract
	Sufficiency C22	Ratio of the confirmed number of fully implemented functions to the number of functions agreed in the service contract
	Reasonable communication mechanism C23	1. Whether is an interactive communication mechanism established and how is it implemented 2. Whether all of the personnel know and understand the communication requirements.
	Compliance C24	1. Service function’s compliance with relevant standards or regulations 2. Ratio of the number of actually observed industry standards with the total number of contracted industry functionality standards
Stability B3	Service Continuity C31	Ratio of average fault-free time with average restoration time
	Service stability C32	Having the ability to ensure continuous and stable delivery of the agreed service level, and having a stable deviation rate agreed in the customer service contract.
	Report timely submission rate C33	Ratio of the number of service reports that are submitted on time to meet the requirements of the service agreement with the number of service reports requested by the service agreement.
Security B4	Permission suitability, C41	Whether access to information and resources can match business requirements
	Information and resource readiness C42	1. Within the agreed service period, whether information and resources can be normally visited or obtained. 2. Ratio of times of accessibility to information and resources normally with total times of information and resource access requested
	Data auditability C43	Ratio of the number of activities with a complete record with the number of activities to be recorded
	Data confidentiality capability of service supplier C44	1. Whether service supplier has established secure strategy and system, and how is it implemented. 2. Whether all of the personnel know and understand the secure strategy and system requirements.

### Extracting the weights and important coefficients of service quality indicators

The weight of each evaluation indicator was calculated. Accordingly, the order of primary indicators, from top to bottom, is professionalism (0.6457), security (0.1193), functionality (0.0958) and stability (0.0596). The top five sub-dimemsions, in sequence, are personnel’s technical ability, hardware attractiveness, data auditability, confidentiality capability, professional service procedures and project compliance (Table 3).

**Table 3:** Weight and prioritization of indicators and sub-indicators using FAHP

Primary indicators	Weight of Primary indicators	Sub-indicators	Weight of sub-indicators	Priority
Professionalism	0.6692	Brand imagine C11	0.0276	7
		Personnel’s technical ability C12	0.2921	1
		Facility and equipment attractiveness C13	0.2378	2
		Professional service procedures C14	0.0882	4
Functionality	0.0958	Integrity of project completion C21	0.0235	9
		Sufficiency of project completion C22	0.016	12
		Reasonable interactive communication mechanism C23	0.0085	14
		Project compliance C24	0.0713	5
Stability	0.0596	Service continuity C31	0.0189	10
		Service stability C32	0.0346	6
		Research report timely submission rate C33	0.0061	15
Security	0.1754	Permission suitability C41	0.0112	13
		Information and resource readiness C42	0.0162	11
		Data auditability C43	0.1208	3
		Data confidentiality capability of service supplier C44	0.0272	8
Total	1.0		1.0	

In this paper, to verify the consistency and validity of expert scoring results, the consistency ratio (CR) was used. CR enables observation of variations between the different pair-wise comparisons. In general, the CR value of the pairwise comparison matrix being less than or equal to 0.1 indicates that the expert’s judgments are reasonable; above 0.1 means weak consistency [40]. The results of this study show that the consistency of expert responses is 0.01, which means that the confidence level is over 90%. It is thus concluded that the responses expressed by the experts are well thought out rather than subjectively determined.

### Service quality grading

For the convenience application, a service quality grading method was developed in this study. First, the experts graded the formula calculation results (FCR) according to the formulas in Table 4. Second, the actual effectiveness value (AEV) of each indicator was achieved by multiplying the FCR times their own weights. Finally, laboratory service ability was graded according to the standards expressed using “star level”. A total of five service quality levels were suggested, in which level 5 was the highest and level 1 was the lowest in the hierarchy (see Table 5 for details). If needed, the level evaluation method can not only evaluate primary indicators, but also independently evaluate secondary indicators.

**Table 4:** Calculation of different index items for “star” grading

Primary indicators	Secondary indicators	Formula	Value Interpretation
Professionalism	Brand imagine C11	X = A/5 A value may equal to 1, 2, 3, 4 or 5 1:unqualified. 2:obtain a lower level qualification. 3:In the top ten of the mainstream of provinces and cities, occupy a larger market share and achieve higher level qualifications. 4:In the top ten of government, industry, manufacturers and users on a national scale, occupy a larger market share and achieve higher level qualifications. 5:Leading in the government, industry, manufacturer and users on a national scale, occupy a larger market share and achieve highest level qualifications.	0 < X ≤ 1, the closer to 1, the better
	Personnel’s technical ability C12	X = X1*70% + X2*30% X1 = A/B A = the number of service personnel who obtain corresponding professional qualification B = the total number of service personnel X2 = C/B C = the number of service personnel who obtain the service related bachelor or above degree	0 ≤ X ≤ 1, the closer to 1, the better
	Facility and equipment attractiveness C13	X = A/5 A value may equal to 1, 2, 3, 4 or 5 1: No tools available 2:Tools are used in some services, but they are less matched 3:Tools are used in some services, and they are well matched 4:Tools are used in all services, but not exactly all matched. 5:Tools are used in all services and fully matched.	0 < X ≤ 1, the closer to 1, the better
	Professional service procedures C14	X = A/5 A value may equal to 1, 2, 3, 4 or 5 1: Neither establish documented service process, the implementation is also very poor 2: No documented service process, but the implementation is good 3: A documented service process was established, but implemented poorly. 4: A documented or automated service process is established and implemented well 5:Passed ISO/IEC 20000 certification and implemented well	0 < X ≤ 1, the closer to 1, the better
Functionality	Integrity of project completion C21	X = A/B A = the actual number of functions implemented B = the number of functions agreed in the service contract	0 ≤ X ≤ 1, the closer to 1, the better
	Sufficiency of project completion C22	X = A/B A = the confirmed number of fully implemented functions B = the number of functions agreed in the service contract	
	Reasonable interactive communication mechanismC23	X = A/5 A value may equal to 1, 2, 3, 4 or 5 1: No established interactive communication mechanism with customers and poorly implemented. 2: No established interactive communication mechanism with customers, but implemented well. 3: The interactive communication mechanism with customer was established, but poorly implemented. 4:The interactive communication mechanism with customer was established, and well implemented. 5:The interactive communication mechanism with customer was established, and well implemented. Furthermore, all of the personnel know and understand the communication requirements.	0 ≤ X ≤ 1, the closer to 1, the better
	Project compliance C24	X = A/B A = The actual number of contracted functionality related industry standards that was met in the service process. B = The total number of contracted functionality related industry standards	0 ≤ X ≤ 1, the closer to 1, the better
Reliability	Service continuity C31	X = A/(A + B) A = average fault-free time B = average restoration time	0 ≤ X ≤ 1, the closer to 1, the better for service continuity
	Service stability C32	$$ {\displaystyle \begin{array}{c}Y=\frac{\sum \limits_{i=1}^n{\left( Xi-\overline{X}\right)}^2}{n}\\ {}\mathrm{X}=1\hbox{-} \mathrm{Y}\end{array}}/\left(\mathrm{Eu}\hbox{-} \mathrm{El}\right) $$ Y: Standard deviation ratio Xi: Sample values for service characteristics X: Sample mean of service characteristics Eu: The upper limit value of the deviation specified in the service agreement El: The lower limit value of the deviation specified in the service agreement n: sampling times for service	If Y > 1,equal to 1; If Y ≤ 1, equal to Y; 0 ≤ X ≤ 1 the closer to 1, the better
	Research report timely submission rate C33	X = A/B A = The number of service reports that are submitted on time to meet the requirements of the service agreement B = Number of service reports requested by the service agreement	0 ≤ X ≤ 1, the closer to 1, the better
Security	Permission suitability C41	X = A/B A = The number of privilege authorized appropriately B = The number of privilege requested by the service agreement	0 ≤ X ≤ 1, the closer to 1, the better
	Information and resource readiness C42	X = A/B A = Times of accessibility to information and resources normally B = Total times of information and resource access requested	0 ≤ X ≤ 1, the closer to 1, the better
	Data auditability C43	X = A/B A = The number of activities with a complete record B = The number of activities to be recorded	0 ≤ X ≤ 1, the closer to 1, the better
	Data confidentiality capability of service supplier C44	X = A/5 A value may equal to 1, 2, 3, 4 or 5 1: No secure strategy and system, and poorly implemented 2:No secure strategy and system, but well implemented 3:Secure strategy and system was established, but poorly implemented 4:Secure strategy and system was established, and well implemented 5:Secure strategy and system was established, and well implemented. Furthermore, all of the personnel know and understand the secure strategy and system requirements.	0 < X ≤ 1 the closer to 1, the better

**Table 5:** the presentation of star rating results

Rating	Actual effective value (X)	Star Level	Markers
Level I	X<0.3	One-Star	★
Level II	0.3 ≤ X<0.5	Two-Star	★★
Level III	0.5 ≤ X<0.7	Three-Star	★★★
Level IV	0.7 ≤ X<0.9	Four-Star	★★★★
Level V	0.9 ≤ X	Five-Star	★★★★★

Note: according to actual effectiveness value, the service quality was represented by one-star (★), two-star(★★), three –star(★★★), four-star(★★★★) or five-star(★★★★★). X = FCR*weights

### Case study

To verify the practical applicability of the proposed service quality evaluation system, a case study was performed. A questionnaire was designed to collect the DMG’s judgments. The DMG’s were then asked to evaluate the service supplier, FW lab, according to the requirements in Table 6.

**Table 6:** Comprehensive evaluation for pre-clinical service quality evaluation of cardiovascular implantation in FW

Primary indicators	Secondary indicators	Actual appraisal Value	Weights	Actual effective Value
Professional	Brand imagine C11	1.00	0.0276	0.0276
	Personnel’s technical ability C12	0.84	0.2921	0.2454
	Facility and equipment attractiveness C13	1.00	0.2378	0.2378
	Professional service procedures C14	0.80	0.0882	0.0706
Functionality	Integrity of project completion C21	0.80	0.0235	0.0188
	Sufficiency of project completion C22	0.70	0.016	0.0112
	Reasonable interactive communication mechanismC23	0.80	0.0085	0.0068
	Project compliance C24	1.00	0.0713	0.0713
Reliability	Service continuity C31	0.6	0.0189	0.0113
	Service stability C32	0.86	0.0346	0.0298
	Research report timely submission rate C33	0.90	0.0061	0.0055
Security	Permission suitability C41	0.80	0.0112	0.0090
	Information and resource readiness C42	0.90	0.0162	0.0146
	Data auditability C43	1.00	0.1208	0.1208
	Data confidentiality capabilityof service supplier C44	1.00	0.0272	0.0272
			Total	0.9076

Given that the FW lab undertakes projects from both clinical researchers and domestic/abroad enterprises, who engage in the research & development of cardiovascular implants devices. Their customers mainly include clinical doctors (project manager), graduates (project executor), and R&D staff of companies. Therefore, in this case study, respondents included different types of customers: clinical doctors (*n* = 10), graduates (n = 10), the company research and development staff (n = 10). A total of 30 questionnaires were handed out, and the recovery number of valid questionnaires was 30 copies. The average age was 35 years old, the sex ratio 3:1 (male: female), all had obtained either a bachelor’s degree, graduate degree or above, and accounted for 75%. According to the survey data and interview contents, the FCR of each index was calculated; combined with primary indicators and sub-indicator’s weight, the comprehensive implementation effectiveness assessment set was obtained. After normalization processing and further assignment, FW’s final comprehensive service quality AEV was 0.9076, therefore, we concluded that the comprehensive service ability of FW for the preclinical development of cardiovascular implant devices is up to five-star level.

## Discussion

Quality is regarded as an important factor in all organizations especially those involving patient life and health. As for the newly arising industry of services for the preclinical development and evaluation of implant devices, there is still no quality evaluation system. In this study, we used the FAHP method to construct a service quality evaluation indicator system, which will provide a method for the service demanders to select ideal suppliers, and for the service suppliers to improve their service quality.

The adopted FAHP technique in this study is one of the most widely used multi-criteria decision making methods [10]. It has been proposed for medical diagnosis, evaluation and selection of medical treatments and therapies; however, no studies have been done with the service quality evaluation for the preclinical development of cardiovascular implant devices. The conventional AHP only takes into account the distinct judgments of decision makers, [18] but it can’t fully reveal human’s fuzzy opinions [16]. So, FAHP method, a fuzzy extension of AHP, was developed by integrating fuzzy comparison ratios. The fuzzy set theory, puts together the comparison process more flexibly and potently in order to clarify experts’ preferences [20]. Practice has proven that it is equally suitable for building a feasible and reasonable service quality evaluation indicator system in our study.

In this study, both customer and service supplier factors were comprehensively considered [41] and a relatively complete indicator system was constructed through FAHP analysis. The indicator system consists of two index hierarchies. Primary dimensions include professionalism, security, functionality and stability. Among them, professionalism is the most important with the highest weight values in the primary indicators, based on our research findings.

Professionalism can be reflected in four aspects: brand image, personnel’s technical ability, facility and equipment attractiveness, and professional service procedures. Brand image refers to the personality characteristics of the company or a certain brand in the market and in the public’s heart. It best reflects the supplier’s professionalism and the public’s recognition of the brand, especially the consumers [42]. However, personnel’s technical ability and facility/equipment attractiveness are the foundation of professionalism [43]. To enhance the brand image and service professionalism, it is an indispensably important content and method to improve personnel’s technical ability and hardwares. With increasingly fierce market competition, professional service procedures have become another core of corporate competition and have become an important strategy for the entire brand [44]. A good sense of service (reputation) can win more customers for the company, which is bound to enhance the market competitiveness of the company.

The top five secondary indicators in the service quality evaluation system include personnel’s technical ability, facility and equipment attractiveness, data auditability, confidentiality capability and professional service procedure in order. Among which, personnel’s technical ability, facility and equipment attractiveness and professional service procedure belong to professionalism as mentioned above. Data auditability and confidentiality capability belong to the primary dimension “security”. Auditability requires a variety of records, which are an important part of the traceability. As for confidentiality, service supplier should establish a secure strategy and system, and all of the personnel should understand the secure strategy and system requirements. This is an important indicator proposed from the perspective of customer requirement.

For the convenience of popularization and application of this system, this study also proposed the use of a “star” system to represent service quality evaluation results. As a case study, this study used the system to assess the service quality of FW lab which is the largest cardiovascular implanted devices preclinical research and development service laboratory in China. The results show that the evaluation results obtained by this system are consistent with the actual survey results.

In terms of enterprise brand image, most of the domestic cardiovascular implant device manufacturers are cooperating with the FW laboratory. FW laboratory has obtained the highest level of qualification, and occupied more than 60% market share. In terms of professional service personnel, the laboratory has a total of 40 employees, all of whom are qualified. More than half of the people have obtained a professional bachelors degree or above, with a solid basis and rich practical experience in team work. FW Laboratory has the first domestic one-stop hybrid operating room dedicated to animal experiment (cleanliness up to grade 10.000). It is equipped with 3 mobile C arm X-ray machines (American GE), the Dutch Philips real-time three-dimensional ultrasound machine, Germany Drager anesthesia machine, a breathing machine and other advanced equipment, which are attractive enough.

Based on the long-term service practice, FW laboratory has accumulated a wealth of experience, mastered advanced technologies and established professional service procedure. The integrity and stability of service, experimental data availability and readiness are satisfactory, which were widely recognized. It is really up to five-star level laboratory. According to the evaluation results, a proposal for improving its service was put forward: because of high frequency use, some precise instruments should be checked regularly to avoid impact on service continuity.

This study has several limitations. Our experts were limited to Beijing shanghai and Tianjin. Future research can be in a more culturally diverse geographical region and compared with the results of this paper since preferences/experiences may change by country, tradition or socioeconomic level. More studies are required to investigate the applicability of the indicators of service quality evaluation system developed in this paper.

## Conclusions

The service quality evaluation system constructed in this study is effective and can be popularized. Application of this system will provide a measurable basis for the service demander to select service supplier and provide a method for the supplier to improve their service quality.

## Additional files

Additional file 1: Expert score sheet. To perform pair-wise comparisons between the first and second levels of indicators, linguistic variables were used in the sheet to convert the measured qualitative factors to fuzzy numbers. EI: equally important; WMI: weakly more important; SMI: strongly more important; VSMI: very strongly more important; AMI; absolutely more important. (PDF 392 kb)

Additional file 2: Raw data for expert scoring. A total of 10 experts were invited to participate in this study. The score sheet recovery rate was 100%. The numbers in the last column represented the average of expert scores. (PDF 85 kb)

Additional file 3: Raw data for the weight and priority of indicators and subindicators using FAHP. With respect to the expert scoring results, the weight and priority of each evaluation indicator were calculated. The order of primary indicators, from top to bottom, was professionalism, security, functionality and stability. The top five sub-dimemsions included personnel’s technical ability, hardware attractiveness, data auditability, confidentiality capability, professional service procedures and project compliance. (PDF 56 kb)

## Abbreviations

AMI: Absolutely more important
CR: Consistency ratio
CVIDs: Cardiovascular implant devices
DMG: Decision-making group
EI: Qually important
FAHP: Fuzzy Analytical Hierarchy Process
PWC: Pair-wise comparison
SMI: Strongly more important
VSMI: Very strongly more important
WMI: Weakly more important
