# Supplementary material for: Construction and application of service quality evaluation system in the preclinical research on cardiovascular implant devices
Source: BMC Med Inform Decis Mak. 2019 Feb 28;19:37. doi: 10.1186/s12911-019-0773-4 (PMC6396521; doi:10.1186/s12911-019-0773-4)
Supplement: Supplementary file 1 — Expert score sheet. To perform pair-wise comparisons between the first and second levels of indicators, linguistic variables were used in the sheet to convert the measured qualitative factors to fuzzy numbers. EI: equally important; WMI: weakly more important; SMI: strongly more important; VSMI: very strongly more important; AMI; absolutely more important. (PDF 392 kb) [file 12911_2019_773_MOESM1_ESM.pdf]

Expert score sheet for cardiovascular implant material preclinical research service quality evaluation

Experts,

In this study, the evaluation index system for preclinical research service quality of cardiovascular devices includes two levels. The first-level index layer includes four dimensions: professionalism, functionality, safety and reliability. Each primary dimensions is divided into several secondary indicators, as detailed in the following table.

Primary dimensions	Secondary dimensions	Secondary indicators
**professionalism**	Brand image of service supplier	The ranking among government, industry, manufacturers and users; the market share; The level of approved qualifications.
	Personnel's technical ability	The ratio of the number of qualified technical personnel to the total number of technical personnel
	Facility and hardware attractiveness	The matching degree between the tools and the project task.
	professional service procedures	Established and practiced standardized service procedures
**Functionality**	Functional integrity	According to the service agreement, completeness of service function being evaluated
	Sufficiency	According to the service agreement, the full extent of the service function being evaluated
	Reasonable communication mechanism	Established an interactive communication mechanism and the status of being implemented
	Compliance	The compliance of service function with relevant standards or regulations
**Stability**	Service Continuity	The ability to ensure that service agreements are met in all circumstances
	Service stability	Ability to provide consistent and stable service to meet agreed standards
	report timely submission rate	Compare the number of service reports that are actually submitted on time to meet service agreement requirements with service agreement requirements
**Security**	Suitability of the customer's available information resources permission settings	Whether access to information and resources can match business requirements
	Information and resource readiness	Within the agreed service period, whether information and resources can be normally visited or obtained.
	Data auditability	Ratio of the number of activities completely recorded to the number of activities to be recorded
	Data confidentiality capability of service supplier	Whether service supplier has established secure strategy and system, and how is it implemented.

Here, please score the importance of indicators influencing the service quality for preclinical research on cardiovascular implant devices by comparing each other. 1/9-9 scaling method was used as the scoring principle which show the relative importance of the former factor compared with the latter factor. Please see the following table for the significance of score scale.

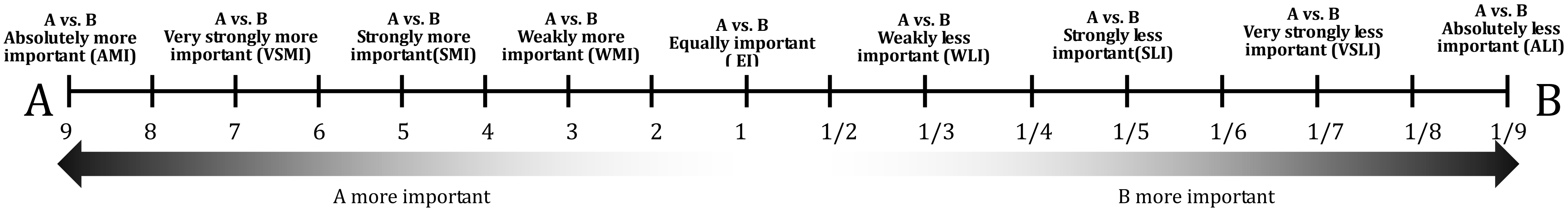

"Example" if you think that "functionality" (A) and "Security" (B) are between WLI and SLI, you are asked to grade:

A	B	The importance in influencing the quality of preclinical research on cardiovascular implants, A vs.B
**functionality**	**Security**	**1/4**

Below, please grade importance of the factors that influence cold chain logistics development in Beijing by comparing each other.

A	B	A vs. B	A	B	A vs. B
**professionalism**	**functionality**		**professionalism**	**Security**	
**professionalism**	**Stability**		**functionality**	**Stability**	
**functionality**	**Security**		**Stability**	**Security**	
Brand image of supplier	Personnel’s technical ability		Brand image of supplier	Facility and equipment attractiveness	
Brand image of supplier	Professional service procedures		Personnel’s technical ability	Facility and equipment attractiveness	
Personnel’s technical ability	Professional service procedures		Facility and equipment attractiveness	Professional service procedures	
Integrity of project completion	Sufficiency of project completion		Integrity of project completion	Reasonable interactive communication mechanism	
Integrity of project completion	Project compliance		Sufficiency of project completion	Reasonable interactive communication mechanism	

Sufficiency of project completion	Project compliance		Reasonable interactive communication mechanism	Project compliance	
**A**	**B**	**A vs. B**	**A**	**B**	**A vs. B**
Service continuity	Service stability		Service continuity	Research report timely submission rate	
Service stability	Research report timely submission rate		Service continuity	Research report timely submission rate	
Permission suitability	Information and resource readiness		Permission suitability	Data auditability	
Permission suitability	Data confidentiality capability of service supplier		Information and resource readiness	Data auditability	
Information and resource readiness	Data confidentiality capability of service supplier		Data auditability	Data confidentiality capability of service supplier	
