# Supplementary material for: Construction and application of service quality evaluation system in the preclinical research on cardiovascular implant devices
Source: BMC Med Inform Decis Mak. 2019 Feb 28;19:37. doi: 10.1186/s12911-019-0773-4 (PMC6396521; doi:10.1186/s12911-019-0773-4)
Supplement: Supplementary file 2 — Raw data for expert scoring. A total of 10 experts were invited to participate in this study. The score sheet recovery rate was 100%. The numbers in the last column represented the average of expert scores. (PDF 85 kb) [file 12911_2019_773_MOESM2_ESM.pdf]

A	B		
		Expert 1	Expert 2
specialism	functionality	5	4
specialism	stability	7	6
specialism	security	3	4
functionality	security	1/4	1/3
functionality	stability	1	1/2
stability	security	1/6	1/6
Brand image of supplier	Personnel’s technical ability	1/6	1/5
Brand image of supplier	Facility and equipment attractiveness	1/3	1/2
Brand image of supplier	Professional service procedures	1/5	1/4
Personnel’s technical ability	Facility and equipment attractiveness	1	1
Personnel’s technical ability	Professional service procedures	5	4
Facility and equipment attractiveness	Professional service procedures	6	7
Integrity of project completion	Sufficiency of project completion	1	1/2
Integrity of project completion	Reasonable interactive communication mecha	2	1
Integrity of project completion	Project compliance	1/4	1/3
Sufficiency of project completion	Reasonable interactive communication mecha	2	1
Sufficiency of project completion	Project compliance	1/4	1/5
Reasonable interactive communication mec	Project compliance	1/7	1/8
Service continuity	Service stability	1/4	1/4
Service continuity	Research report timely submission rate	2	1
Service stability	Research report timely submission rate	3	4
Permission suitability	Information and resource readiness	6	6
Permission suitability	Data auditability	1/6	1/6
Permission suitability	Data confidentiality capability of service supp	1/6	1/4
Information and resource readiness	Data auditability	1/4	1/5
Information and resource readiness	Data confidentiality capability of service supp	1/4	1/4
Data auditability	Data confidentiality capability of service supp	5	4

A vs. B								
Expert 3	Expert 4	Expert 5	Expert 6	Expert 7	Expert 8	Expert 9	Expert 10	Average
5	6	4	3	5	7	6	5	5
5	8	7	7	6	8	7	8	6.9
3	2	2	3	5	4	3	3	3.2
1/2	1/5	1/3	1/4	1/2	1/4	1/3	1/5	0.315
2	1	1	1/2	1/2	2	1	1	1.05
1/5	1/7	1/6	1/7	1/5	1/6	1/7	1/6	0.1661905
1/6	1/7	1/5	1/6	1/7	1/5	1/6	1/5	0.1752381
1	1/2	1/4	1/3	1/2	1/4	1/3	1/4	0.425
1/2	1/5	1/3	1/4	1/3	1/5	1/3	1/5	0.28
1/2	1	1/2	1/2	2	2	1	1	1.05
4	5	3	5	3	2	6	6	4.3
7	6	5	4	4	5	7	6	5.7
1/2	2	2	1	1/2	2	1	1/2	1.1
2	2	3	3	1	1	2	3	2
1/4	1/3	1/3	1/5	1/4	1/5	1/5	1/4	0.26
2	2	1	3	3	3	2	1	2
1/3	1/4	1/3	1/4	1/4	1/3	1/5	1/5	0.26
1/6	1/7	1/8	1/6	1/7	1/5	1/7	1/6	0.1521429
1/3	1/5	1/4	1/4	1/3	1/5	1/5	1/3	0.26
3	2	2	3	2	1	1	3	2
3	2	2	4	3	5	4	3	3.3
7	4	6	8	7	5	5	6	6
1/5	1/6	1/7	1/8	1/4	1/6	1/5	1/6	0.175119
1/5	1/6	1/7	1/6	1/6	1/5	1/7	1/7	0.1745238
1/6	1/4	1/4	1/4	1/2	1/5	1/6	1/2	0.2733333
1/5	1/4	1/6	1/5	1/2	1/4	1/4	1/6	0.2483333
6	5	6	4	5	3	5	4	4.7
