# Supplementary material for: Construction and application of service quality evaluation system in the preclinical research on cardiovascular implant devices
Source: BMC Med Inform Decis Mak. 2019 Feb 28;19:37. doi: 10.1186/s12911-019-0773-4 (PMC6396521; doi:10.1186/s12911-019-0773-4)
Supplement: Supplementary file 3 — Raw data for the weight and priority of indicators and subindicators using FAHP. With respect to the expert scoring results, the weight and priority of each evaluation indicator were calculated. The order of primary indicators, from top to bottom, was professionalism, security, functionality and stability. The top five sub-dimemsions included personnel’s technical ability, hardware attractiveness, data auditability, confidentiality capability, professional service procedures and project compliance. (PDF 56 kb) [file 12911_2019_773_MOESM3_ESM.pdf]

A	B
1. professionalism	functionality
professionalism	Stability
functionality	Security
Brand image of supplier	Personnel’s technical ability
Brand image of supplier	Professional service procedures
Personnel’s technical ability	Professional service procedures
Integrity of project completio	Sufficiency of project completion
Integrity of project completio	Project compliance
Sufficiency of project complet	Project compliance

A	B
Service continuity	Service stability
Service stability	Research report timely submission rate
Permission suitability	Information and resource readiness
Permission suitability	Data confidentiality capability of service suppli
Information and resource readi	Data confidentiality capability of service suppli

TABLE 3 Weight and prioritization of indicators and sub-indicators using FAHP.

Primary indicators	Weight of Primary indicators
professionalism	0.6457
functionality	0.1193
Stability	0.0596
Security	0.1754

A vs. B	A
	professionalism
	functionality
	Stability
	Brand image of supplier
	Personnel' s technical ability
	Facility and equipment attractiveness
	Integrity of project completion
	Sufficiency of project completion
	Reasonable interactive communication mechanism
A vs.B	A
	Service continuity
	Service continuity
	Permission suitability
.er	Information and resource readiness
.er	Data auditability

0.0596
0.6457

Sub-indicators	Weight of sub-indicators
Brand imagine C11	0.0276
Personnel' s techni	0.2921
Facility and equipm	0.2378
Professional servic	0.0882
Integrity of projec	0.0235
Sufficiency of proj	0.016
Reasonable interact	0.0085
Project compliance	0.0713
Service continuity	0.0189
Service stability C	0.0346
Research report tim	0.0061
Permission suitabil	0.0112
Information and res	0.0162
Data auditability C	0.1208
Data confidentialit	0.0272

B	A vs. B
Security	
Stability	
Security	
Facility and equipment attractiveness	
Facility and equipment attractiveness	
Professional service procedures	
Reasonable interactive communication mechanism	
Reasonable interactive communication mechanism	
Project compliance	
B	A vs. B
Research report timely submission rate	
Research report timely submission rate	
Data auditability	
Data auditability	
Data confidentiality capability of service supplier	

0.1754
0.1193

Priority	
	7
	1
	2
	4
	9
	12
	14
	5
	10
	6
	15
	13
	11
	3
	8
